# Relationship between the Occurrence of Genetic Variants of Single Nucleotide Polymorphism in microRNA Processing Genes and the Risk of Developing Multiple Sclerosis

**DOI:** 10.3390/biomedicines10123124

**Published:** 2022-12-03

**Authors:** Justyna Basak, Danuta Piotrzkowska, Ireneusz Majsterek, Ewa Kucharska

**Affiliations:** 1 Department of Clinical Chemistry and Biochemistry, Medical University of Lodz, Mazowiecka 5, 92-215 Lodz, Poland; 2 Department Geriatrics and Social Work, Jesuit University Ignatianum in Cracow, Kopernika 26, 31-501 Cracow, Poland

**Keywords:** multiple sclerosis, microRNA, single nucleotide polymorphism

## Abstract

Multiple sclerosis (MS) is an autoimmune demyelinating disorder of the central nervous system (CNS), which leads to disturbances in the conduction of nerve impulses, cognitive impairment, sensory and motor disturbances, as well as depressive symptoms. MS remains an incurable disease with a difficult diagnosis and unclear etiology. The aim of the analysis was to identify SNPs that may potentially be associated with an increased risk of developing MS. Blood samples were obtained from patients with MS (194 subjects) and age-matched healthy controls (188 subjects). The polymorphic variant frequencies of rs197412 T>C in *GEMIN3*, rs7813 G>A in *GEMIN4*, rs1106042 G>A in *HIWI*, rs10719 A>C in *DROSHA*, rs3742330 A>G in *DICER1*, rs11077 T>G in *XPO5*, rs14035 C>T in *RAN*, rs636832 G>A in *AGO1* were determined in DNA using real-time PCR TaqMan^®^ SNP Genotyping Assay. Our findings indicate that the GG *AGO1* rs636832 and AA *GEMIN4* rs7813 genotypes were associated with an increased risk of MS. Although our findings provide a clearer understanding of the pathogenesis of MS, further investigations are needed to better understand their potential for the evaluation of other miRNA processing genes believed to be associated with MS etiology.

## 1. Introduction

Multiple sclerosis (MS) is an autoimmune demyelinating disorder of the central nervous system (CNS) that is characterized by multifocal lesions within white and gray matter. The disease causes extensive damage to the myelin sheaths around the axons, which leads to disturbances in the conduction of nerve impulses, and may manifest in cognitive impairment, sensory and motor disturbances, as well as depressive symptoms. Although MS can take several different forms, the most common type is relapsing-remitting MS (RRMS), characterized by alternating periods of remission and exacerbation of symptoms [1,2]. MS most often affects young people aged 20–40 and is the most significant cause of disability among this age group, contributing to high economic burdens and widespread social consequences. According to global epidemiological data, the number of people suffering from MS continues to grow, and is currently estimated at 2.8 million, with a two-fold dominance of cases in women. Despite the development of further therapeutic methods, MS remains an incurable disease with a difficult diagnosis, especially in the early stages, and an unclear etiology. Its development is believed to be influenced by both environmental and genetic factors [2,3].

MicroRNAs (miRNAs) are small, non-coding single-stranded RNAs, typically about 22 nucleotides long, which regulate gene expression. It is estimated that 30% of human genes are modulated by the activity of specific miRNAs; therefore, defects in their biogenesis may represent a significant pathological factor [4,5]. Deregulation of miRNAs may influence the development of neuroinflammatory processes and stimulate the differentiation of immune cells that favor autoimmunity [5]. Although a growing body of evidence suggests that many miRNAs are involved in the pathogenesis of MS, particularly miR-146, miR-155, miR-223, and miR-326, the reasons for their deregulation remain unclear [6]. The expression of mature miRNAs can be modulated by several mechanisms, including epigenetic modifications, transcription factor activity, and the activity and levels of proteins influencing the processing of miRNA transcripts [7]. Primary miRNA (pri-miRNA) are degraded by the DROSHA/DGCR8 complex into hairpin precursor miRNA (pre-miRNA). Pre-miRNAs are transported from the transcription site, i.e., the cell nucleus, to the cytoplasm via exportin-5 (XPO5)/RAN complex, where they are further processed by DICER, HIWI, GEMIN3, GEMIN 4 and Argonaute 1–4 (AGO) proteins [8,9,10]. Previous studies have revealed significant increases in the expression of miRNA processing proteins such as DROSHA, DICER and DGCR8 in blood samples of patients with RRMS compared to healthy individuals [11]. Hence, functionally relevant single nucleotide polymorphisms (SNPs) located within the sequence of genes encoding miRNA processing proteins may significantly influence the development of MS. The aim of the present in silico analysis was to identify SNPs that may potentially be associated with an increased risk of developing MS.

## 2. Materials and Methods

A total of 194 patients with RRMS (Table 1) and 188 healthy controls were recruited from the Neurological Rehabilitation Division, III General Hospital in Lodz and the Vadimed Medical Center in Krakow, Poland. MS patients were diagnosed according to the lates McDonald’s criteria (2017 version). The study was approved by the Commission of Bioethics at the Medical University of Lodz. All qualified subjects gave their written consent to participate in the study. Patients with severe psychiatric illness, cancer or other neurological, autoimmune or inflammatory disorders were excluded from the trial. In addition, participants aged below 18 years and above 70 years, and those who found it difficult to make verbal contact were also excluded. The control group comprised those not diagnosed with MS or other acute diseases, including cancer and neurodegenerative disorders. The control group was adjusted to the study group in terms of age and sex. Before the study began, all participants underwent a medical examination.

### 2.1. Selection of SNPs

The NCBI dbSNP SNP database (https://www.ncbi.nlm.nih.gov/snp/, accessed on 13 May 2020)) was searched for polymorphisms located within the sequence of key miRNA processing genes (i.e., *DROSHA*, *DICER1*, *XPO5*, *RAN*, *AGO1*, *GEMIN3*, *GEMIN4* and *HIWI*) that could potentially be genetic markers for MS in the European population (minor allele frequency (MAF) > 0.05). A similar search was also made of the literature data. SNPs located in the coding (rs197412 T>C in *GEMIN3*, rs7813 G>A in *GEMIN4*, rs1106042 G> A in *HIWI*) and non-coding regions that could potentially affect the level of gene expression (rs10719 A>C in *DROSHA*, rs3742330 A>G in *DICER1*, rs11077 T>G in *XPO5*, rs14035 C>T in *RAN*, rs636832 G> A in *AGO1*) were selected for further study.

### 2.2. Genotyping

Peripheral blood was collected from patients and controls into EDTA, and subjected to DNA isolation using commercially-available kits for DNA extraction (Blood Mini, A&A Biotechnology, Gdansk, Poland). The genotyping of SNP was performed by using the real-time PCR and TaqMan™ SNP Genotyping Assay (Applied Biosystems™, ThermoFisher Scientific, Waltham, MA, USA), which contains primers and TaqMan minor groove binder (MGB) probes specific for each SNP, as well as TaqMan™ Universal Master Mix II, no UNG (Applied Biosystems™, ThermoFisher Scientific, Waltham, MA, USA) according to the manufacturer’s protocol. The real-time PCR was performed using a CFX Connect Real-Time PCR Detection System (Bio-Rad, Hercules, CA, USA). For the genotyping of the selected SNPs, 50 ng of extracted DNA was added to a final amount of 10 μL reaction mixture containing 5 μL master mix and 0.5 μL primer/probe mix) and nuclease-free water (DEPC treated). Real-time PCR conditions were as follows: the initial denaturation step was performed at 95 °C for 10 min, then 40 cycles were performed consisting of a denaturation at 95 °C for 15 s and an annealing and elongation step at 95 °C for 60 s. Each trial was performed in two independent replications.

### 2.3. Reverse Transcription Quantitative PCR (RT-qPCR)

Gene expression was evaluated on one group of 30 MS patients and another of 30 healthy controls. Total RNA was isolated from peripheral blood using a RiboPure™ RNA Purification Kit (Invitrogen™, ThermoFisher Scientific, Waltham, MA, USA). The isolated RNA was reverse-transcribed using a High-Capacity cDNA Reverse Transcription Kit (Applied Biosystems™, ThermoFisher Scientific, Waltham, MA, USA) according to the manufacturer’s protocol. 400 ng of RNA was used each time for the reverse transcription reaction. The expression of *AGO1* and *GEMIN4* genes was determined using real-time PCR with primers complementary to the tested sequences (*GEMIN4*: Fow. ACT GTA AAG TCC AGG CTG CTR, Rev.CCA CGT GGT CAA ACT CCT CTG T, *AGO1*: Fow. TGA CAG CCA AGG TAT CTT TCC TTA, Rev.TGA GTG TGG GTA TCT AAA ATC TCT GT; *GAPDH*: Fow. TGC CCA GTT GAA CCA GGG G, Rev. CGC GGA GGG AGA GAA CAG TGA), PowerUp™ SYBR™ Green Master Mix (Applied Biosystems™, ThermoFisher Scientific, Waltham, MA, USA) and CFX Connect Real-Time PCR Detection System (Bio-Rad, Hercules, CA, USA). Real-time PCR conditions were as follows: uracil-DNA glycosylase (UDG) activation was performed at 50 °C for 2 min, initial denaturation was performed at 95 °C for two minutes, then 40 cycles were performed consisting of a denaturation at 95 °C for 15 s, primer annealing at 64 °C for 15 s, and an elongation step at 72 °C for 60 s. Each trial was performed in two independent replications and *GAPDH* was used as the reference gene. The level of relative gene expression was assessed by the ΔCt method.

### 2.4. Statistical Analysis

A statistical analysis was performed using Statistica 13.1 software (StatSoft, Tulsa, OK, USA). To determine the relationship between the occurrence of the studied SNPs and the risk of developing MS, a logistic regression analysis was performed, and the odds ratio (OR) was calculated for the occurrence of genotypes in the study and control groups (95% CI). To analyze the distribution of variants, the Hardy-Weinberg Equilibrium (HWE) was used, which was evaluated using the goodness-of-fit Chi-square test. To assess the level of significance of expression comparison in the study and control group, a non-parametric test (U Mann-Whitney) was used. The distribution of variables was assessed by the Shapiro–Wilk test. *p* values less than 0.05 were considered statistically significant.

## 3. Results

Among the eight selected SNPs (*DROSHA* rs10719, *XPO5* rs11077, *RAN* rs14035, *AGO1* rs636832, *DICER1* rs3742330, *GEMIN3* rs197412, *GEMIN4* rs636832, *HIWI* rs1106042), four SNPs (i.e., *DROSHA* rs10719, *XPO5* rs11077, *RAN* rs14035 and *GEMIN3* rs197412) were found to be noncompliant with the Hardy-Weinberg law (*p* > 0.05) and were excluded from further analysis. The general characteristics and the distribution of genotypes of the analyzed SNPs in the study group and the control group are presented in Table 2 and Table 3. We selected SNPs that have functional potential. In addition to SNPs located in the coding region (GEMIN4 rs7813 and HIWI rs1106042), which directly affect the change in the amino acid sequence of the protein, we also selected SNPs located in non-coding regions that may affect the regulation of gene expression and protein conformation, such as in the case of intronic AGO1 rs636832, which can affect protein structure and functionality by altering mRNA splicing. In turn, DICER1 rs3742330 is in the 3′-UTR region and may play a significant role in the regulation of gene expression, transcript stability and may also affect the miRNA binding site.

Statistically significant differences were found between the occurrence of GA and GG genotypes of *AGO1* rs636832, as well as between the AA and AG of *GEMIN4* rs7813. The GG *AGO1* rs636832 and AA *GEMIN4* rs636832 genotypes were associated with an increased risk of MS (OR = 1.8218, 95% CI, 1.0336–3.2108; *p* = 0.0350 and OR = 2.2588, 95% CI, 1.3940–3.6602; *p* = 0.0007 respectively), while the GAAGO1 rs636832 and GA GEMIN4 rs636832 genotypes were associated with a lower risk of developing MS (OR = 0.5025; 95% CI; 0.2778–0.9089; *p* = 0.0202 and OR = 0.4479; 95% CI; 0.2962–0.6775; *p* = 0.0001 respectively). For the *AGO1* gene (rs636832), the frequency distributions of the AA genotype were comparable in the two groups, while those of the A and G alleles were slightly different between controls and patients, with A predominating in the control group and G in the patients; however, no statistical significance was demonstrated. Also, for the *GEMIN4* alleles (rs636832), the frequency of the GG genotype and A and G alleles were similar between the groups, with A demonstrating a slight tendency to dominate in the patient group. No association was found between the *DICER1* (rs3742330) and *HIWI* (rs1106042) polymorphisms and the risk of MS, although for *HIWI* (rs1106042), the frequencies of GA and AA and of allele A were lower in MS patients than in the control group.

### 3.1. Allele and Genotype Combinations Analysis

An allele-allele combination and genotype combination analysis was performed for four SNPs (*AGO1* rs636832, *GEMIN4* rs7813, *DICER1* rs3742330, *HIWI* rs1106042) to assess the synergic effect of these SNPs on the risk of MS (Table 4). All of the combinations tested were associated with a lower risk of MS. The analysis of allele combinations in four SNPs (for *AGO1/GEMIN4/DICER1/HIWI* combination) revealed significant differences between the patient and control groups for the following allele sets: G-G-A-G (OR = 0.5364; 95% CI; 0.3384–0.8504; *p* = 0.0072), A-G-A-G (OR = 0.5180; 95% CI; 0.2783–0.9641; *p* = 0.0343) and A-A-A-G (OR = 0.4734; 95% CI; 0.2482–0.9028; *p* = 0.0198).

The three-allelic model revealed differences in similar combinations for *AGO1/GEMIN4/DICER* i.e., G-G-A (OR = 0.4863; 95% CI; 0.3044–0.7767; *p* = 0.0021), A-A-A (OR = 0.4552; 95% CI; 0.2395–0.8654; *p* = 0.0136) and A-G-A (OR = 0.4986; 95% CI; 0.2687–0.9253; *p* = 0.0242), as well as for *AGO1/GEMIN4/HIWI*, i.e., G-G-G (OR = 0.5028; 95% CI; 0.3156–0.8010; *p* = 0.0033), A-A-G (OR = 0.4734; 95% CI; 0.2482–0.9028; *p* = 0.0198), A-G-G (OR = 0.5180; 95% CI; 0.2783–0.9462; *p* = 0.0343); however, for the *GEMIN4/DICER1/HIWI* combination, only one set was statistically significant: G-A-G (OR = 0.5268; 95% CI; 0.3301–0.8405; *p* = 0.0064).

In the case of biallelic models, significant differences were noted for the three combinations of alleles of the *AGO1/GEMIN4* set–G-G (OR = 0.4540; 95% CI 0.2825–0.7297; *p* = 0.0009), A-A (OR = 0.4552; 95% CI 0.2395–0.8654; *p* = 0.0136) and A-G (OR = 0.4986; 95% CI 0.2687–0.9253; *p* = 0.0242). Differences were also observed for A-A (OR = 0.5489; 95% CI 0.3116–0.9671; *p* = 0.0350) for *AGO1/DICER1*, G-A (OR = 0.4757; 95% CI 0.2955–0.7657; *p* = 0.0018) for *GEMIN4/DICER1* and G-G (OR = 0.4925; 95% CI 0.3068–0.7904; *p* = 0.0028) for *GEMIN4/HIWI*. Among the combinations of genotypes, only the GA *AGO1* and GA *GEMIN4* heterozygotes showed a synergistic effect on the risk of MS (OR = 0.3714; 95% CI; 0.1720–0.8018; *p* = 0.0082).

### 3.2. AGO1 and GEMIN4 Expression Analysis

**Figure 1:**
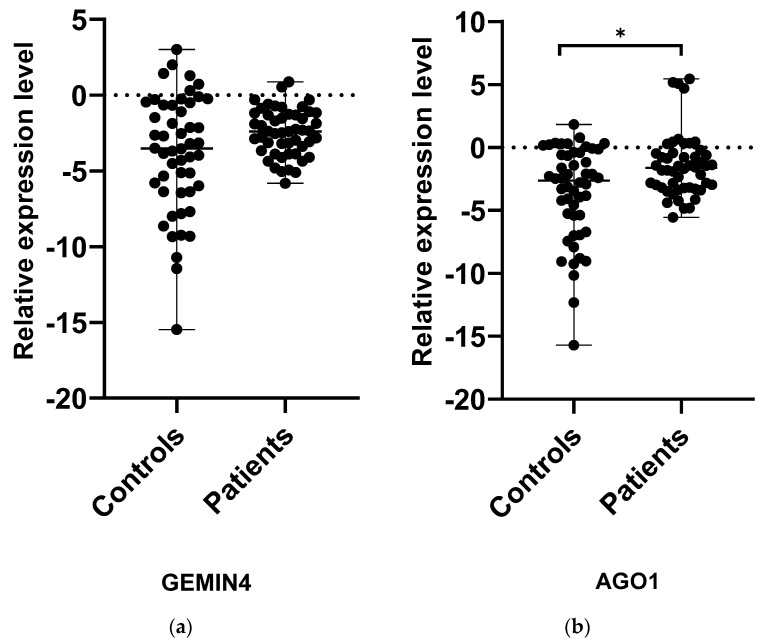
Expression levels of *GEMIN4* (**a**) and *AGO1* (**b**) in PBMCs from MS patients and healthy controls. Statistical analysis of differences between the groups of data was carried out using the Mann–Whitney U-test (* indicate statistical significance at a *p* < 0.05).

**Figure 2:**
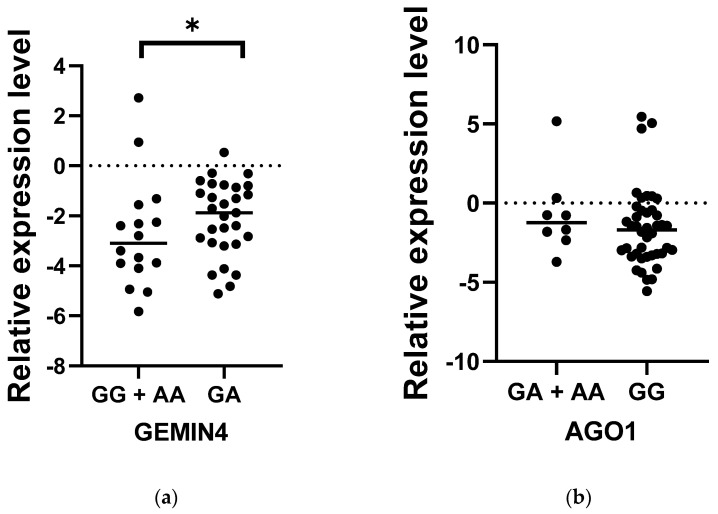
Comparison of the expression level of *GEMIN4* rs7813 (**a**) and *AGO1* rs636832 (**b**) genetic variants in the group of patients with MS. Statistical analysis of differences between the groups of data was carried out using the Mann–Whitney U-test (* indicate statistical significance at a *p* < 0.05).

Among all the examined genes, AGO1 and GEMIN4 were selected to assess whether the level of expression in patients with MS may deviate from the norm; this could indicate that the SNPs have some functional significance. While the relative level of AGO1 expression in MS patients was slightly higher compared to controls (*p* < 0.05), no significant differences were found between the study and control groups in the case of GEMIN4 (*p* > 0.05) (Figure 1). Interestingly, in the case of both genes, the level of expression demonstrated wider dispersal in the control group than the patient group, in which individual subjects demonstrated more similar expression levels. To thoroughly analyze the relationship between the occurrence of the SNPs AGO1 rs636832 and GEMIN4 rs7813 and the level of expression of their genes, the study compared their relative levels of expression between MS patients carrying the genotypes GG, GA and AA (Figure 2). In the case of GEMIN4 rs7813, the most numerous group are patients with the GA genotype: the expression of the GEMIN4 gene is significantly greater among heterozygotes than homozygotes, i.e., GG and AA (*p* < 0.05). However, for AGO1, no statistically significant differences in expression were found between individual genotypes (*p* > 0.05).

## 4. Discussion

The present study evaluates the miRNA processing pathway as a potential influence on the development of MS. Abnormal miRNA expression is believed to contribute to many common human diseases, including neurodegenerative diseases. Previous research has focused on genetic variants within miRNA targets or within miRNA genes; as such, the relationship between the SNPs of microRNA biosynthetic genes and the risk of MS has not been extensively studied. The present study is the first such study to focus on SNPs within miRNA biosynthesis genes associated with a greater risk of MS. Any resulting disturbances in miRNA processing caused by the SNP can inhibit the formation of mature miRNAs and disturb their function. This may influence the level of gene and protein expression, which is crucial in maintaining homeostasis and can lead to neurodegenerative disease [7,12].

This study examined the polymorphic variants of genes known to be involved in miRNA biogenesis (*DROSHA* rs10719, *XPO5* rs11077, *RAN* rs14035, *AGO1* rs636832, *DICER1* rs3742330, *GEMIN3* rs197412, *GEMIN4* rs7813, *HIWI* rs1106042) in patients with MS and in a control group. Four SNPs (viz. *DROSHA* rs10719, *XPO5* rs11077, *RAN* rs14035 and *GEMIN3* rs197412) did not comply with the Hardy-Weinberg law (*p* > 0.05) and were excluded from further analysis.

Two SNPs, *GEMIN4* (rs7813) G>A (R [Arg] > C [Cys]) and *HIWI* (rs110604) G>A (R [Arg] > K [Lys]) are located in exon regions, and can hence cause changes in the amino acid sequence of the protein. In contrast, *AGO1* rs636832 G>A is located in the intron, which may affect the mRNA splicing process and possibly result in the creation of an abnormal transcript. Finally, *DICER1* (rs3742330) A>G is situated in the 3ʹ UTR, which could potentially affect the efficiency of miRNA biogenesis.

DICER is one of the key enzymes involved in miRNA biogenesis, which cleaves the characteristic loop structure of a pre-miRNA to form mature miRNAs [13]. Research on DICER expression in MS patients is inconclusive; Jafari et al. indicate that patients with RRSM demonstrate more than twice the level of DICER compared to healthy subjects. Other reports, however, indicate that DICER expression is selectively downgraded in B cells [11,14,15]. Although the genetic variation in the DICER gene may explain expression deregulation, our findings do not indicate any relationship between rs3742330 and the occurrence of MS, and the issue requires further analysis.

*HIWI* is a relatively poorly understood gene belonging to the Ago protein-related PIWI family encoding an endoribonuclease which is part of the RISC complex. The research to date on its role in the pathogenesis of human diseases focused mainly on cancer [16,17,18]. Our research does not confirm any relationship between the rs1106042 polymorphism located within this gene and the presence of MS. Nevertheless, this does not exclude the possibility that HIWI may be involved in the process of MS development through another mechanism.

GEMIN4 and AGO1 belong to the group of proteins involved in the selective binding of the guide strand and the formation of the RISC, which recognizes the mRNA 3′-UTR sequences and causes translational repression of the target transcript [19]. Although it seems that GEMIN4 and AGO1 may be significantly involved in the deregulation of miRNA silencing and processing, they have not yet been examined in MS patients. Our findings indicate that the GG *AGO1* rs636832 and AA *GEMIN4* rs7813 genotypes were associated with an increased risk of MS, while GA *AGO1* rs636832 and GA *GEMIN4* rs7813 were associated with a lower risk of MS. The analysis of the frequency of combinations of the *AGO1* rs636832 and *GEMIN4* rs7813 genotypes suggests that the heterozygotes of these SNPs appear to cooperate in reducing the risk of developing MS.

Interesting data was also provided by analyzing the frequencies of combinations of alleles of the studied SNPs: specific sets of single alleles were found to be much more common in healthy individuals, even if the same alleles were more common in MS patients in the analysis of frequencies for single genes. The results suggest that some variants may to some extent suppress the effect of alleles that favor the development of MS. However, these findings should be interpreted with particular caution as the genotype combination analysis did not reveal any significant differences between the test and control groups, with the exception of the GA/GA for *AGO1* rs636832/*GEMIN4* rs7813 combination.

Promising results were obtained from our analysis of the expression of the two genes *AGO1* and *GEMIN4*, indicating that the presence of the SNP is associated with the occurrence of MS. Until now, research into the expression of miRNA processing genes in MS has focused on the *DROSHA*, *DICER1* and *DGCR8* genes [11]. Our present findings are the first to demonstrate that the level of *AGO1* is elevated in PBMC in MS patients. In the case of the *GEMIN4* gene, no significant difference in expression was found between the groups, possibly due to the small size of the study group selected for expression analysis. These results suggest that there may be a relationship between the occurrence of the rs636832 polymorphism in *AGO1* and the level of expression of this gene, although the SNP is localized within the intron. It is known that such intronic SNPs could be associated with human diseases and may also affect splicing, which in turn could affect the expression level [20,21]. Additionally, although no difference in *GEMIN4* expression was found between the MS patients and controls, *GEMIN4* was overexpressed in patients who were heterozygous for the *GEMIN4* SNP rs7813; further research is needed to determine how the genetic variants of miRNA processing genes regulate their expression and how they influence the development of MS.

Few studies have examined the polymorphic variation of miRNA processing genes and their association with the risk of MS. Moreover, there is no data concerning the role of these SNPs in other neurodegenerative diseases. One report has investigated the SNP variants of *RAN* rs14035 and *GEMIN3* rs197388 and their possible influence on the risk of Alzheimer’s Disease (AD); however, the authors did not find any association between these SNPs and the risk of AD development [22]. One report found SNP rs3742330 of the DICER gene to be associated with the development of MS [23].

The SNP variations of *GEMIN4* and *AGO1* have been found to influence different types of cancer. SNP rs7813 of the *GEMIN4* gene could induce Arg to Cys substitution at the 1033 amino acid position through C to T transition. Horikawa et al. found this SNP to be associated with a reduced risk of renal cell carcinoma [24]. Liang et al. placed rs7813 at the top of a list of 226 microRNA biosynthesis gene SNPs associated with ovarian cancer risk in a Caucasian population [25]. In addition, the CT heterozygotes and T allele carriers of *RAN* rs14035 were found to have a lower risk of colorectal cancer [26].

Our present findings were obtained from a Caucasian population of Polish ethnicity; due to the variation in polymorphic MS between populations, it may be difficult to compare them with those of other ethnic groups. Nevertheless, our study provides new information concerning SNP variations in the miRNA processing genes *AGO1* and *GEMIN4*, and their possible implications in the pathogenesis of MS. Our correlation of genotype frequencies provides a wider view of MS progression. An increasing number of reports highlighting the role of miRNA in neurodegeneration [3,5,6,7] suggests that the polymorphic variants of genes involved in miRNA biogenesis may play significant roles in the neurodegeneration processes occurring during MS. However, further investigation is needed to confirm our findings and support any potential strategy for early diagnosis of MS.

Our findings indicate that the GG *AGO1* rs636832 and AA *GEMIN4* rs7813 genotypes were associated with an increased risk of MS. This is the first report to evaluate the role of SNPs of miRNA processing genes in the development of MS. Although our findings provide a clearer understanding of the pathogenesis of MS, further investigations are needed to more fully understand their potential for the evaluation of other miRNA processing genes believed to be associated with MS etiology. For a deeper insight into the significance of miRNA processing genes in the pathogenesis of MS, further research directions should focus on analyzing the frequency of SNPs and the expression of miRNA processing genes in other ethnic groups and including different subtypes of MS, which, along with our findings, may contribute to the development of MS prognostic tests, as well as to the improvement of the differential diagnosis with other neurological diseases, especially those with a similar clinical picture.

## Figures and Tables

**Table 1:** Clinical characteristics of RRMS patients.

Characteristic of Study Group	Total
Number of patients	194
Age (years, $\bar{x}$ ± s)	54.2 ± 12.9
Expanded disability status scale (EDSS) (scale 1–10) ^1^	
0–5.5	147 (75.8%)
6–6.5	29 (14.9%)
>7	18 (9.3%)
Modified Rankin Scale (mRS) (scale 1–5) ^2^	
0–1	99 (51.0%)
2	48 (24.7%)
3	29 (14.9%)
4	14 (7.2%)
5	4 (2.0%)

^1^ 0–5.5—no or little impairment to walking; 6–6.5—requires one or two walking aids; >7—wheelchair mobility or confined to bed. ^2^ 0–1—lack of disability; 2—slightly disabled; 3—average degree of disability; 4—fairly severe degree of disability; 5—very severe degree of disability.

**Table 2:** General characteristics of the analyzed SNPs of miRNA processing genes.

Gene	SNP	Position	Region	Major/Minor Allele	Amino Acid Change	MAF ^1^	MAF (Control/Patients)
*AGO1*	rs636832	chr1:35897874	intron	G>A		0.0807	0.1037/0.0670
*GEMIN4*	rs7813	chr17:744946	exon	G>A	R (Arg) > C (Cys)	0.5604	0.5027/0.5541
*DICER1*	rs3742330	chr14:95087025	3′-UTR	A>G		0.0907	0.0824/0.0851
*HIWI*	rs1106042	chr12:130357093	exon	G>A	R (Arg) > K (Lys)	0.0611	0.0691/0.0422

^1^ minor allele frequency (MAF) in the European population.

**Table 3:** The frequency distribution of the genetic variants of the rs636832, rs7813, rs3742330 and rs1106042 polymorphisms and their relationship with the risk of developing MS.

Genotype/ Allele	Control (N = 188)		Patients (N = 194)		Odds Ratio (OR) (95% CI)	*p*
	Number	Frequency	Number	Frequency		
NC_000001.11:g.35897874G>A—*AGO1* (rs636832)						
A/A	2	0.0106	3	0.0155	1.4607 (0.2399–8.8948)	0.6771
**G/A**	**35**	**0.1862**	**20**	**0.1031**	**0.5025** **(0.2778–0.9089)**	**0.0202**
**G/G**	**151**	**0.8032**	**171**	**0.8814**	**1.8218** **(1.0336–3.2108)**	**0.0350**
**χ^2^ = 2.1581; *p* = 0.3399**						
A	39	0,1037	26	0.0670	0,6188 (0,3683–1,0396)	0.0666
G	335	0,8910	362	0.9330	1,6209 (0,9641–2,7252)	0.0650
NC_000017.11:g.744946G>A—*GEMIN4* (rs7813)						
**A/A**	**33**	**0.1755**	**63**	**0.3247**	**2.2588** **(1.3940–3.6602)**	**0.0007**
**G/A**	**123**	**0.6543**	**89**	**0.4588**	**0.4479** **(0.2962–0.6775)**	**0.0001**
G/G	32	0.1702	42	0.2165	1.3470 (0.8069–2.2487)	0.2518
**χ^2^ = 4.9333; *p* = 0.4712**						
A	189	0.5027	215	0.5541	1.2296 (0.9248–1.6349)	0.1544
G	187	0.4973	173	0.4459	0.8133 (0.6155–1.0816)	0.1542
NC_000014.9:g.95087025A>G—*DICER1* (rs3742330)						
A/A	159	0.8457	162	0.8351	0.9233 (0.5277–1.6155)	0.7754
G/A	27	0.1436	31	0.1598	1.1341 (0.6476–1.9862)	0.6595
G/G	2	0.0106	1	0.0052	0.4819 (0.0430–5.4007)	0.5404
**χ^2^ = 0.0453; *p* = 0.9776**						
A	345	0.9176	355	0.9150	0.9666 (0.5888–1.5870)	0.8966
G	31	0.0824	33	0.0851	1.0345 (0.6188–1.7296)	0.8966
NC_000012.12:g.130357093G>A—*HIWI* (rs1106042)						
A/A	3	0.0160	1	0.0052	0.3195 (0.0327–3.1223)	0.2896
G/A	20	0.1064	15	0.0773	0.3897 (0.2081–0.7297)	0.3244
G/G	165	0.8777	178	0.9175	1.5508 (0.7899–3.0445)	0.1974
**χ^2^ = 7.2224; *p* = 0.0270**						
A	26	0.0691	17	0.0422	0.6168 (0.3286–1.1578)	0.1277
G	350	0.9309	371	0.9578	1.6212 (0.8619–3.0492)	0.1277

**Table 4:** Analysis of the synergistic effect of a combination of genetic variants of the rs636832, rs7813, rs3742330 and rs1106042 polymorphisms on the risk of developing MS.

Allele Combinations	Controls	Patients	Odds Ratio (OR) (95% CI)	*p*
*AGO1* G>A/*GEMIN4* G>A/*DICER1* A>G/*HIWI* G>A				
G-G-A-G	148	129	0.5364 (0.3384–0.8504)	0.0072
A-G-A-G	31	18	0.5180 (0.2783–0.9641)	0.0343
A-A-A-G	30	16	0.4734 (0.2482–0.9028)	0.0198
*AGO1* G>A/*GEMIN4* G>A/*DICER1* A>G				
G-G-A	151	129	0.4863 (0.3044–0.7767)	0.0021
A-A-A	31	16	0.4552 (0.2395–0.8654)	0.0136
A-G-A	32	18	0.4986 (0.2687–0.9253)	0.0242
*AGO1* G>A/*GEMIN4* G>A/*HIWI* G>A				
G-G-G	150	129	0.5028 (0.3156–0.8010)	0.0033
A-A-G	30	16	0.4734 (0.2482–0.9028)	0.0198
A-G-G	31	18	0.5180 (0.2783–0.9462)	0.0343
*GEMIN4* G>A/*DICER1* A>G/*HIWI* G>A				
G-A-G	150	131	0.5268 (0.3301–0.8405)	0.0064
*AGO1* G>A/*GEMIN4* G>A				
G-G	153	129	0.4540 (0.2825–0.7297)	0.0009
A-A	31	16	0.4552 (0.2395–0.8654)	0.0136
A-G	32	18	0.4986 (0.2687–0.9253)	0.0242
*AGO1* G>A/*DICER1* A>G				
A-A	37	23	0.5489 (0.3116–0.9671)	0.0350
*GEMIN4* G>A/*DICER1* A>G				
G-A	153	131	0.4757 (0.2955–0.7657)	0.0018
*GEMIN4* G>A/*HIWI* A>G				
G-G	152	131	0.4925 (0.3068–0.7904)	0.0028
*AGO1* G>A/*GEMIN4* G>A				
GA/GA	24	10	0.3714 (0.1720–0.8018)	0.0082

## Data Availability

Not applicable.
